# Routing Functions for Parameter Space Decomposition to Describe Stability Landscapes of Ecological Models

**DOI:** 10.1007/s11538-025-01554-7

**Published:** 2025-11-14

**Authors:** Joseph Cummings, Kyle J.-M. Dahlin, Elizabeth Gross, Jonathan D. Hauenstein

**Affiliations:** 1 Department of Applied and Computational Mathematics and Statistics, University of Notre Dame, Notre Dame, IN 46556 United States; 2 Department of Mathematics and Center for the Mathematics of Biosystems, Virginia Tech, Blacksburg, VA 24061 United States; 3 Department of Mathematics, University of Hawai‘i at Mānoa, Honolulu, HI 96822 United States

**Keywords:** Real numerical algebraic geometry, algebraic biology, stability analysis, competition-colonization, coral-bacteria symbiosis

## Abstract

Changes in environmental or system parameters often drive major biological transitions, including ecosystem collapse, disease outbreaks, and tumor development. Analyzing the stability of steady states in dynamical systems provides critical insight into these transitions. This paper introduces an algebraic framework for analyzing the stability landscapes of ecological models defined by systems of first-order autonomous ordinary differential equations with polynomial or rational rate functions. Using tools from real algebraic geometry, we characterize parameter regions associated with steady-state feasibility and stability via three key boundaries: singular, stability (Routh-Hurwitz), and coordinate boundaries. With these boundaries in mind, we employ routing functions to compute the connected components of parameter space in which the number and type of stable steady states remain constant, revealing the stability landscape of these ecological models. As case studies, we revisit the classical Levins-Culver competition-colonization model and a recent model of coral-bacteria symbioses. In the latter, our method uncovers complex stability regimes, including regions supporting limit cycles, that are inaccessible via traditional techniques. These results demonstrate the potential of our approach to inform ecological theory and intervention strategies in systems with nonlinear interactions and multiple stable states.

## Introduction

Biological transitions, while ubiquitous in nature, are often exacerbated or caused by human activities. Many of the most consequential events experienced by people or societies are biological transitions – including tumor development, disease outbreaks, and ecosystem collapse. A greater understanding of the causes of these transitions allows us to better detect, prevent, manage, or revert them. Table 1 provides some examples of biological systems, transitions that they may undergo, and possible causes.

Transitions occur due to changes to key aspects of these systems (possibly small in magnitude and duration), leading to large-magnitude, long-term changes in the system. These transitions may be due to perturbations to system parameters – for example, eliminating malaria by decreasing the reproduction rate of mosquitoes – or by short-term changes in the system state caused by external forces – such as one-time immigration of infected individuals causing a disease outbreak. Mathematically, these transitions can be described by assessing the *stability* of the equilibrium states of the system. In order to study system transitions, we must first model the system in its current state – often represented as a steady-state (though no real system is ever truly at equilibrium). This is generally done by mechanistically describing the processes that cause changes in the system state as functions of the current system state. However, determining the existence and stability of steady states may sometimes be a non-trivial mathematical challenge even when the functions take the form of relatively simple polynomials or rational functions. A major goal is to determine how the *stability landscape* shifts as parameter values are perturbed. These parameter perturbations can correspond to interventions aimed at curtailing transitions or enabling them in order to achieve a desired state.

**Table 1:** Transitions of biological systems and their possible causes

System	Initial state	Altered state	Possible cause
Cells	Healthy	Diseased	Shift in mutation rates
Cells	Diseased	Healthy	Medication
Drylands	Semi-arid	Desert	Increased grazing pressure
Lake	Healthy	Eutrophic	Increased temperatures
Population	Disease-free	Epidemic	Introduction of infected individual
Population	Endemic disease	Disease-free	Vaccination
Population	Abundant	Extinct	Increased mortality due to predation
Population	Sparse	Abundant	Translocation intervention

In this paper, we set out to apply a recent algebraic geometry method to determine the stability landscape of any dynamical system described by a system of first-order autonomous ordinary differential equations, ${\dot{x}} = f\left( x\right)$ where the rate function *f* is polynomial or rational in *x*. Models of this form are ubiquitous in the mathematical biology literature and often used to describe population dynamics, the spread of disease within a population, or the spread of infection among a population of cells. With the use of *routing functions* (Cummings et al. 2025), we are able to decompose parameter space into (not necessarily disjoint) connected components within which certain equilibria exist and are stable. Furthermore, regions of overlap among the connected components indicate where multistability is possible, that is, where perturbations to the system state (not the parameter values), can lead to transitions.

As a case study, we consider a model representing the dynamics of a host and two of its symbionts that is described by a system of ODEs with rate functions given by multivariate polynomials of degree two. While these functions are simple, the full stability analysis of this model is intractable employing commonly-used techniques. To make the analysis tractable, we fix all but four parameters and demonstrate using routing functions to analyze slices of the stability landscape when the coral is intrinsically viable and when the coral is intrinsically non-viable. As a by-product of this analysis, we also determine the existence of a region of parameter space wherein the model must exhibit limit cycles simply by determining the complement of the identified connected components. Our hope is that this new method can be useful for mathematical biologists analyzing similarly formulated systems.

The rest of the article is organized as follows. Section 2 summarizes steady-state analysis and describes the proposed approach using routing functions. This approach is applied to the Levins-Culver model (Levins and Culver 1971) in Section 3 to reproduce known results. Section 4 applies the approach to a tripartite symbiosis system. A short conclusion is provided in Section 5.

## Steady-State Analysis

This paper is concerned with understanding the steady states of a dynamical system with polynomial or rational rate function. To this end, consider the dynamical system

$$\begin{aligned}{\dot{x}} = f(x;a)\end{aligned}$$

where $a = (a_1, \ldots , a_k) \in {\mathbb {R}}_{>0}^k$ are parameters, $x=(x_1, \ldots , x_n) \in {\mathbb {R}}_{\ge 0}^n$ are variables and $f = (f_1, \ldots , f_n)$ are real-valued polynomial or rational functions. For a given *a*, the point *x* is a *steady-state* of the system if $f(x;a) = 0$. A steady-state *x* is *locally asymptotically stable*, or simply *stable*, if the eigenvalues of the Jacobian matrix of *f* with respect to the variables evaluated at *x*, denoted $J_xf(x;a)$, all have negative real parts. Otherwise, *x* is *unstable*.

Each point *a* in the parameter space will give rise to a certain number of real positive steady-states and a certain number of stable steady-states. In fact, the parameter space ${\mathbb {R}} _{>0}^k$ can be decomposed into regions where these counts are constant. For a polynomial dynamical system as described above, the boundaries of these regions are determined by an algebraic hypersurface (possibly reducible) called the *discriminant locus* (described in Section 2.1), and the regions themselves are semi-algebraic sets. Thus, determining these regions is fundamentally a real algebraic geometry problem. See, e.g., Bernal et al. (2023); Gelfand et al. (2008); Lazard and Rouillier (2007) for more details.

Determining these regions of parameter space are of much interest in mathematical modeling. Regions of the parameter space that give rise to multiple real-positive steady-states are often referred to as *regions of multistationarity*, while regions of the parameter space that give rise to multiple real-positive stable steady-states are often referred to as *regions of multistability*. A concrete understanding of these regions can aid in model selection and discrimination (Feliu and Wiuf 2013). In some cases, just determining whether regions of multistationarity or regions of multistability exist for a dynamical system can be a challenging question (Conradi et al. 2017; Joshi and Shiu 2015).

As an illustration of the challenge to determine these regions of parameter space, consider the following model that represents a predator population *y* with a foraging rate that follows a Holling Type II functional response and a prey population *x* with a strong Allee effect:

1 $$\begin{aligned} \begin{aligned} \frac{dx}{dt}&= x\left( 1 - \frac{x}{K}\right) \left( \frac{x}{A} - 1\right) - \frac{\alpha xy}{1 + \beta x}, \\ \frac{dy}{dt}&= y\left( -m + \frac{\gamma x}{1 + \beta x}\right) . \end{aligned} \end{aligned}$$

The parameters of this system are $\alpha$, $\beta$, $\gamma > 0$. The right-hand side is rational where the denominators do not vanish on the sets of interest. In fact, there are four steady-states: total extinction (0, 0), two prey-only steady-states, $\left( A,0\right)$ and $\left( K,0\right)$, and a coexistence steady-state, $(x^{*}, y^{*})$, provided that $x^{*} = \frac{m}{\gamma - m\beta }>0$ and

2 $$\begin{aligned} y^* = \frac{(1+\beta x^*)(K-x^*)(x^*-A)}{\alpha A K}> 0 ~~\Longrightarrow ~~ (K-x^*)(x^*-A)>0. \end{aligned}$$

Note that total extinction is stable with eigenvalues $-1$ and $-m$. Assume that

$$\begin{aligned}0<K<x^*=\frac{m}{\gamma -m\beta }<A.\end{aligned}$$

Then, $\left( A,0\right)$ is unstable and $\left( K,0\right)$ is stable. Since the system is only two-dimensional, the stability of the coexistence steady-state $(x^{*}, y^{*})$ can be determined simply by computing the signs of the trace and determinant of the Jacobian matrix evaluated at $(x^{*}, y^{*})$, say $J^*$:

$$\begin{aligned} \begin{aligned} \text {tr}(J^*)&=\left( 1-\frac{x^{*}}{K}\right) \left( \frac{x^{*}}{A}-1\right) \left( \frac{1+2\beta x^{*}}{1+\beta x^{*}}\right) +\left( 1-\frac{x^{*}}{K}\frac{x^{*}}{A}\right) ,\\ \det (J^*)&=\left( \frac{\gamma }{\left( 1+\beta x^{*}\right) ^{2}}\right) x^{*}\left( 1-\frac{x^{*}}{K}\right) \left( \frac{x^{*}}{A}-1\right) . \end{aligned} \end{aligned}$$

The positivity of $\det (J^*)$ is straightforward via $x^*>0$ and (2). Thus, the coexistence steady-state is stable if $\text {tr}(J^*)<0$ and the corresponding stability boundary arises from $\text {tr}(J^*)=0$.

If we fix all parameters except one, we can use $\text {tr}(J^*)$ to straightforwardly determine the stability of the coexistence steady-state. Suppose that $(\alpha ,\beta ,\gamma ,m,K) = (2,3/2,1,1/2,1)$. Now, for example, if $A= 9/4$, then $(x^*,y^*) = (2,2/9)$ is stable with $\text {tr}(J^*)=-7/12<0$ and $\det (J^*)=1/72>0$. When $A = 30/11$, then $(x^*,y^*)=(2,8/15)$ has $\text {tr}(J^*)=0$ and thus lies on the boundary of stability. Furthermore, as one increases *A* to pass through the boundary, for example at $A = 3$, then $(x^*,y^*)=(2,2/3)$ and $\text {tr}(J^*)=1/4>0$ so that $(x^*,y^*)$ is unstable. But extending this analysis to higher dimensions, say by allowing *K* to also be a free parameter, leads to a much more difficult problem.

In this work, which is demonstrated using two models from ecology, we use techniques based on routing functions (described in Section 2.2) to compute the decomposition of the parameter space. A full description of the decomposition of the parameter space is called a *landscape*, or *parameter landscape*. We will focus primarily on the *stability landscape* for our systems of interest. In the stability landscapes described below, the number and type (e.g., coexistence or exclusion) of stable steady states are constant in each region.

### Boundaries

As mentioned above, for a polynomial or rational dynamical system, the region boundaries of a parameter landscape are contained in an algebraic hypersurface. The following describes how to obtain this hypersurface in general. To aid the description, we use language from computational algebraic geometry, e.g., see Cox et al. (2015) for a general background. For simplicity, we make some assumptions about the system *f* that hold throughout. Given a generic $a^*$, we assume that the system $f(x;a^*) = 0$ has a finite number of isolated solutions, say *d*, over the complex numbers, all of which are nonsingular, i.e., $f(x;a^*)=0$ implies $\det (J_xf(x;a^*))\ne 0$. Moreover, we assume that, for all *a*, the system $f(x;a)=0$ has exactly *d* solutions counting multiplicity.

In considering how to compute the region boundaries, we need to first consider the different ways that the number of real positive stable steady-states can change as we vary the parameters *a*. For our application, this will result in three types of algebraic boundaries: the *singular boundary*, the *stability (Routh-Hurwitz) boundary*, and *coordinate boundaries*. The singular boundary, which is the classical discriminant locus, arises from parameters *a* where there exists *x* with $f(x;a) = 0$ and $\det (J_xf(x;a))=0$. The stability (Routh-Hurwitz) boundary arises when the greatest real part among all eigenvalues (the spectral abscissa of $J_xf(x;a))$, becomes 0. Finally, a coordinate boundary arises when a coordinate of a steady-state becomes 0.

**Singular boundary** The first way that the number of real positive stable steady-states can change is by the *total* number of real solutions changing. This can only occur when $f(x;a)=0$ has a singular solution, i.e., $\det (J_xf(x;a))=0$. The (Euclidean) closure of the set of such parameters *a*, called the *singular boundary*, forms an algebraic subset of the parameter space. For an illustrative example, consider the quadratic equation

$$\begin{aligned}f(x;a)=a_1 x^2 + a_2 x + a_3 = 0.\end{aligned}$$

Thus, the singular boundary arises when

$$\begin{aligned}f(x;a)=a_1x^2 + a_2x + a_3 = 0 ~~\hbox {and}~~ f'(x;a) = 2a_1 x + a_2 = 0\end{aligned}$$

which, upon eliminating *x*, yields the classical discriminant locus defined by

$$\begin{aligned} D(a) = a_2^2 - 4a_1a_3 =0. \end{aligned}$$

In particular, $f(x;a)=0$ has two real solutions when $D(a)>0$, no real solutions when $D(a)<0$, and one real solution (with multiplicity two) when $D(a)=0$.

As illustrated in the quadratic above, the singular boundary arises as the zero set of an *elimination ideal*, which can be computed algorithmically using Gröbner bases. Since elimination ideals are key to this perspective computationally, we will present the boundaries, including the singular boundary, through an ideal-theoretic perspective. The first piece is the *equilibrium ideal* ${\mathcal {I}}_f = \langle f \rangle \subset \mathbb {C}[x,a]$. The corresponding algebraic set is the *equilibrium variety*

$$\begin{aligned} {\mathcal {V}}_f = {\mathcal {V}}({\mathcal {I}}_f) = \{ (x, a) \in {\mathbb {C}}^{n+k}~|~f(x;a) = 0\}.\end{aligned}$$

Notice that both the parameters *a* and the variables *x* are being treated as indeterminates in this setup. The projection map $\pi _a: {\mathbb {C}}^{n+k} \rightarrow {\mathbb {C}}^{k}$ defined by $\pi _a(x,a)=a$ is the canonical projection onto the parameter space. The geometric analog to the algebraic operation of elimination is projection.

With the assumptions above, the *ideal of the singular boundary* is defined by

$$\begin{aligned} \left( {\mathcal {I}}_f + \langle \det (J_xf)\rangle \right) \cap \mathbb {C}[a]\end{aligned}$$

and the *singular boundary* is the corresponding algebraic set. For example, via Gröbner bases, there is an algorithmic approach to compute the ideal of the singular boundary.

Especially for biological and ecological systems, the equilibrium ideal ${\mathcal {I}}_f$ need not be prime, i.e., the equilibrium variety ${\mathcal {V}}_f$ is reducible. For example, the system in (1) has four prime components. When ${\mathcal {I}}_f$ is not prime, one can construct the ideal of the singular boundary for each prime component, which is typically an easier computation.

**Stability (Routh-Hurwitz) boundary** The second way that the number of real positive stable steady-states can change is by a change in the *stability* of a steady-state, i.e., when the real part of an eigenvalue becomes 0. Since we will use the Routh-Hurwitz criterion (Hurwitz 1895) to determine stability, we will refer to this boundary as the *Routh-Hurwitz boundary*. Since the singular boundary arises from an eigenvalue becoming 0, the *Routh-Hurwitz boundary* contains the singular boundary.

Given parameters *a*, let *x* be a corresponding steady-state. A necessary Routh-Hurwitz condition for *x* to be stable is that the coefficients of the characteristic polynomial of $J_xf(x;a)$, namely, $\det (\lambda I - J_xf(x;a))$, are all positive. In particular, when *f* is polynomial or rational, there are polynomials or rational functions $c_n(x;a),c_{n-1}(x;a),\dots ,c_0(x;a)$ with

$$\begin{aligned} \det (\lambda I - J_xf(x;a)) = c_n(x;a)\lambda ^n + c_{n-1}(x;a) \lambda ^{n-1} + \cdots + c_0(x;a).\end{aligned}$$

Note that $c_n(x;a) = 1$, $c_{n-1}(x;a) = -\text {tr}(J_xf(x;a))$, and $c_0(x;a) = (-1)^n \det (J_xf(x;z))$. Hence, the necessary condition is that $c_i(x;a) > 0$ for $i=0,\dots ,n$.

With $c_n(x;a)=1$, the sufficient Routh-Hurwitz condition is that the associated Hurwitz matrix of the characteristic polynomial must be positive definite (see Appendix A). The Hurwitz matrix is constructed from the coefficients $c_i(x,a)$ of the characteristic polynomial, and we can detect positive-definiteness by considering the leading principal *i*-minors, $\Delta _i$, of *H*. We call each $\Delta _i$ for $i=1,\dotsc ,n$ a *Routh polynomial*. An alternative description of this criterion is through the so-called *Routh array* which is fully described in Dorf and Bishop (2011), while the Routh array and the Hurwitz matrix are described in Gantmacher (1959, Vol. 2, Chapter XV)

Since the systems in this paper either have $n=2$ or $n=3$, we explicitly provide the corresponding conditions where $c_n(x;a)=1$. For $n=2$, the Routh-Hurwitz criterion for stability is simply that the two Routh polynomials, namely $c_1(x;a)$ and $c_0(x;a)$, are positive. This corresponds with $\text {tr}(J_xf(x;a)) < 0$ and $\det (J_xf(x;a)) > 0$ as utilized in the preliminary example at the start of the section.

For $n=3$, the Routh-Hurwitz criterion for stability is that the following three Routh polynomials are positive:

$$\begin{aligned}c_2(x;a),~~ c_2(x;a)c_1(x;a) - c_3(x;a)c_0(x;a), ~~c_0(x;a).\end{aligned}$$

With $c_3(x;a)=1$, positivity of these three Routh polynomials imply $c_1(x;a)>0$.

The *Routh-Hurwitz boundary* is the (Euclidean) closure of the set of parameters *a* such that $f(x;a)=0$ and some Routh polynomial vanishes. With $c_n(x;a)=1$, there are *n* Routh polynomials, say $r_1(x;a), \dots , r_n(x;a)$. Thus, for $R(x;a) = \prod _{i=1}^n r_i(x;a)$, the *ideal of the Routh-Hurwitz boundary* is defined by

$$\begin{aligned}\left( {\mathcal {I}}_f + \langle R\rangle \right) \cap \mathbb {C}[a].\end{aligned}$$

As with the singular boundary, one can construct the ideal of the Routh-Hurwitz boundary for each prime component of ${\mathcal {I}}_f$ and each Routh polynomial.

**Coordinate boundary** Finally, the third way that the number of real positive stable steady-states can change is if a steady-state is no longer positive. Given a variable, say $x_j$, the $x_j$*-boundary* is the Zariski closure of the set of all parameter values where the system $f(x;a)=0$ has a solution with $x_j=0$. Similar, to the singular boundary and the Routh-Hurwitz boundary, the *ideal of the *$x_j$*-boundary* is $\left( {\mathcal {I}}_f + \langle x_j\rangle \right) \cap \mathbb {C}[a]$.

As in the illustrative example (1) and as will be seen in Sections 3 and 4 , it could be the case that certain types of steady-states have some coordinates identically equal to zero, e.g., extinction and prey-only in the illustrative example above. In such cases, the corresponding ideal for the coordinate boundaries would be the zero ideal, $\langle 0\rangle$, since every parameter value *a* has a solution with that coordinate equal to zero. Thus, to obtain relevant information associated with coordinate boundaries, one needs to consider each prime component of ${\mathcal {I}}_f$ separately and assess which coordinate boundaries are relevant for each prime component.

Putting everything together and removing redundancies, one obtains the *total boundary*. The framework described above is flexible in that one can consider the total boundary for the entire system or only for certain types of steady-states. Nonetheless, with the assumptions above, the total boundary is either empty or is a union of hypersurfaces, called a *hypersurface arrangement*, in $\mathbb {C}^k$. Since an empty total boundary is not interesting, we assume that the total boundary, ${\mathcal {B}}\subset \mathbb {C}^k$, is a hypersurface arrangement of the form

3 $$\begin{aligned} {\mathcal {B}} = \bigcup _{i=1}^m \{a~|~g_i(a)=0\} \end{aligned}$$

where $g_1,\dots ,g_m$ are nonconstant polynomials in *a*. In particular, the type and number of stable steady-states remain constant on each connected component of $\mathbb {R}_{>0}^k\setminus {\mathcal {B}}$. The last step is to compute such connected components, which is considered next.

### Routing Function and Connected Components

The following describes how to gain an understanding of the connected components of $\mathbb {R}_{>0}^k\setminus {\mathcal {B}}$ using *routing functions*, which are functions that have been used to study connectivity of real semi-algebraic sets (Cummings et al. 2025; Hong 2010; Hong et al. 2020). We note here that in the next section, we will take ${\mathcal {B}}$ as in (3); however, in this section, we allow ${\mathcal {B}}$ to be any hypersurface arrangement in $\mathbb {R}_{>0}^k$. For a full accounting of routing functions and justification for the algorithm proposed in this section, we refer the reader to Cummings et al. (2025). To provide a more comfortable introduction to routing functions, we will use a running example where $k = 2$.

#### Example 1

(Two Ellipses) As our running example, we will consider the complement of a hypersurface arrangement: $\mathbb {R}^2_{>0} \setminus {\mathcal {B}}$ with ${\mathcal {B}}$ defined by the vanishing of the two polynomials below.

$$\begin{aligned} g_1&= (a_1-5)^2 + 4(a_2 - 5)^2 - 16 \\ g_2&= 4(a_1 - 5)^2 + (a_2 - 5)^2 - 16 \end{aligned}$$

**Fig. 1:**
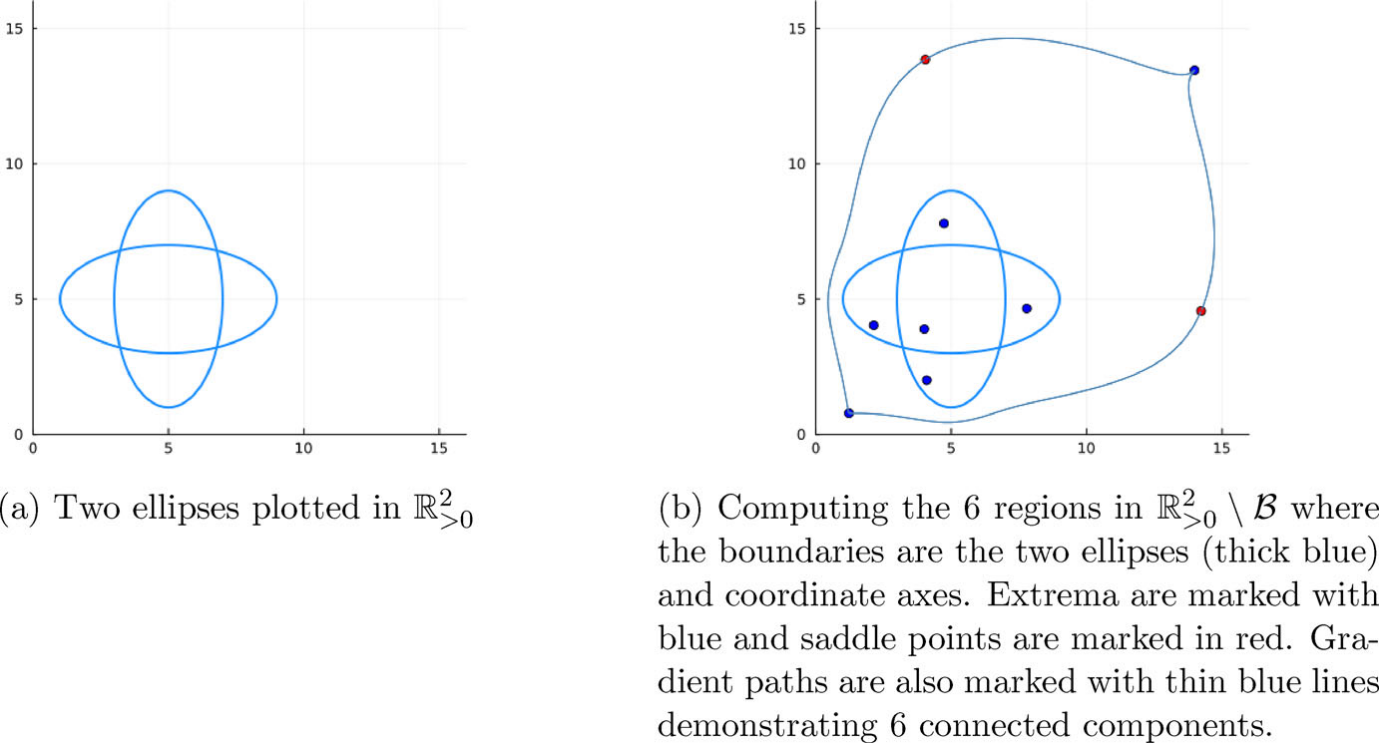
Illustration of the two ellipses and associated region decomposition (Color figure online)

As illustrated in Figure 1a, the set $\mathbb {R}^2_{>0} \setminus {\mathcal {B}}$ has six connected components. We will use a routing function to determine the connected components.

With the general case in mind where ${\mathcal {B}}$ is the zero set of a collection of polynomials $g_1(a), \ldots , g_m(a)$ in indeterminates $a_1, \ldots , a_k$, let us consider the following rational function:

4 $$\begin{aligned} r_{c}(a) = \frac{g_1(a)\cdots g_m(a) \prod _{i=1}^k a_i}{(1 + (a_1-c_1)^2+\cdots +(a_k-c_k)^2)^D} \end{aligned}$$

where $$c\in \mathbb {R}_{>0}^k$$ and $$2D > k + \sum _{i=1}^m \deg (g_i)$$.

For a generic choice of $c \in \mathbb {R}_{>0}^k$, the function $r_{c}$ satisfies the following conditions (Cummings et al. 2025, Thm. 3.4):

1. For all $\epsilon > 0$, there exists $\delta >0$ so that if $\Vert x\Vert >\delta$, then $|r_{c}(x)| < \epsilon$.
2. There are finitely many critical points of $r_{c}$, i.e., solutions of $\nabla r_{c}(a) = 0$, on $\mathbb {R}_{>0}^k \setminus {\mathcal {B}}$.
3. For each critical point $a \in \mathbb {R}_{>0}^k \setminus {\mathcal {B}}$ of $r_{c}$, the Hessian of $r_{c}$ evaluated at *a*, namely $Hr_{c}(a)$, is invertible, i.e., each critical point is non-degenerate.
4. For each $\alpha \in \mathbb {R}\setminus \{0\}$, there is at most one critical point *a* of $r_{c}$ satisfying $r_{c}(a) = \alpha$.

Such a function $r_{c}$ is called a *routing function* for $\mathbb {R}_{>0}^k \setminus {\mathcal {B}}$ and the critical points of $r_{c}$ in $\mathbb {R}_{>0}^k \setminus {\mathcal {B}}$ are called *routing points*. The *index* of a routing point *a* is the number of eigenvalues of $Hr_{c}(a)$ which have the same sign as $r_{c}(a)$. Hence, the index 0 routing points are precisely the local maxima when $r_{c}>0$ and local minima when $r_{c}<0$. An eigenvector *v* of $Hr_{c}(a)$ of unit length with a corresponding eigenvalue $\lambda$ that has the same sign as $r_{c}(a)$ is said to be an *unstable eigenvector* with *v* and $-v$ being the corresponding *unstable eigenvector directions*.

We note that formulation in (4) differs slightly from Cummings et al. (2025). Here we have added the product of the variables to the numerator. This allows us to restrict our attention to just the positive orthant as, in this article, we are only interested in positive parameter values.

#### Example 2

(Two Ellipses Continued) For a generic choice of $c\in \mathbb {R}^2_{>0}$, we can construct a routing function of form (4); this function will help us find information about the six connected components of $\mathbb {R}^2_{>0} \setminus {\mathcal {B}}$. For our experiment, we will use $c = (1.2, 0.7)$. Our routing function is

$$ r_{(1.2,0.7)}(a_1,a_2) = \frac{g_1\cdot g_2 \cdot a_1\cdot a_2}{(1 + (a_1 - 1.2)^2 + (a_2 - 0.7)^2)^4}. $$

Note that the numerator has degree 6 and the denominator has degree 8; thus, this function is bounded. Moreover, the denominator never vanishes on $\mathbb {R}^2$; thus, $r_c$ has no poles, and only vanishes on the coordinate axes and on ${\mathcal {B}}$. With these insights, we can observe that $r_c$ will have at least one critical point in each connected component of $\mathbb {R}^2_{>0}\setminus {\mathcal {B}}$.

In fact, $r_c$ has 9 critical points in $\mathbb {R}^2_{>0}\setminus {\mathcal {B}}$. Of these critical points 7 are local extrema, and 2 are saddle points. The 7 local extrema are plotted in red and the 2 saddle points are plotted in blue in Figure 1. There is an obvious problem in that there are more critical points than connected components. We will now talk about how this can be remedied.

One outcome of Theorem 4.4 of Cummings et al. (2025) is that connected components of $\mathbb {R}^k_{>0}\setminus {\mathcal {B}}$ are in one-to-one correspondence with the connected sets of routing points where the connections arise from gradient ascent/descent along unstable eigenvector directions as follows. If *a* is a routing point with index > 0 and *v* is an unstable eigenvector direction, consider the following initial value problem:

5 $$\begin{aligned} {\left\{ \begin{array}{ll} {\dot{y}}(t) = \textrm{sign}(r_{c}(a)) \cdot \nabla r_{c}(y), \\ \lim _{t\rightarrow 0^+}\frac{{\dot{y}}(t)}{\Vert {\dot{y}}(t)\Vert } = v, \\ \lim _{t \rightarrow 0^+}y(t) = a. \end{array}\right. } \end{aligned}$$

By Proposition 4.2 in Cummings et al. (2025), the trajectory *y*(*t*) is well-defined and $\lim _{t\rightarrow \infty } y(t)$ must also be a routing point. Looping over all routing points and all unstable eigenvector directions yields the relevant connections between the routing points.

It is natural to construct a graph whose vertices are the routing points of $r_{c}$ in $\mathbb {R}_{>0}^k\setminus {\mathcal {B}}$ and whose edges arise by the connections between the routing points via 5. In fact, Algorithm 1 computes such a graph with the following justified by Theorem 4.4 in Cummings et al. (2025).

#### Theorem 2.1

The connected components of the graph *G* computed by Algorithm 1 are in one-to-one correspondence with the connected components of $\mathbb {R}^k_{>0}\setminus {\mathcal {B}}$.

#### Example 3

(Two Ellipses Continued) The fact that $r_c$ has more critical points than connected components can be remedied by following gradient paths emanating away from the saddle points. To be precise, if *P* is a saddle point of $r_c$ and $r_c(P) > 0$, then there is an eigenvector *v* of the Hessian of $r_c$ evaluated at *P* pointing in the direction in which $r_c$ increases. We then consider the two points $P \pm \epsilon v$ for small epsilon and track these points using the gradient flow $\nabla r_c$ which must land at a local maximum in the same connected component. If $r_c(P) < 0$, then we find the eigenvectors *v* corresponding to directions where the function decreases and follow $-\nabla r_c$ which must eventually land at a local minimum in the same connected component. In Figure 1, these gradient paths are marked with thin blue lines.

Now, if we consider a combinatorial graph *G* where the vertices are the critical points of $r_c$ and the edges are the gradient paths, we can identify the connected components of $\mathbb {R}^2_{>0}\setminus {\mathcal {B}}$ with the connected components of the graph *G*.

Since ${\mathcal {B}}$ is a hypersurface arrangement, there is a recently developed Julia package called HypersurfaceRegions.jl (Breiding et al. 2024) that can be used to perform the computations in Algorithm 1.

**Algorithm 1:**
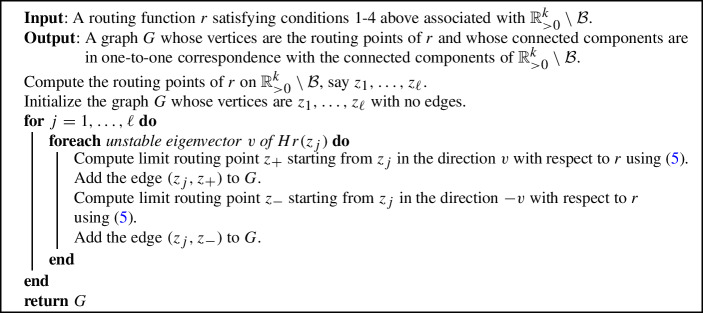
Connected Components

Given a point $a\in \mathbb {R}^k_{>0}\setminus {\mathcal {B}}$ which is not a routing point, the output of Algorithm 1 can be used to determine which connected component of $\mathbb {R}^k_{>0}\setminus {\mathcal {B}}$ contains *a*. For this, one simply solves the following initial value problem:

6 $$\begin{aligned} {\left\{ \begin{array}{ll} {\dot{y}}(t) = \textrm{sign}(r_{c}(a)) \cdot \nabla r_{c}(y), \\ y(0) = a. \end{array}\right. } \end{aligned}$$

By Proposition 4.1 in Cummings et al. (2025), the trajectory *y*(*t*) is well-defined and $\lim _{t\rightarrow \infty } y(t)$ must be a routing point in the same connected component as *a*.

The computationally-intensive parts of this approach lie in computing the total boundary symbolically and computing the routing points numerically–especially when the degree of $r_c$ is large. As the size of the system increases, these parts become challenging on both symbolic and numerical computations. Reducing the number of parameters by fixing some to constant values is a common approach to reduce the computational burden to obtain some insights.

## Levins and Culver Model of Competition-Colonization Trade-Off

Scientists have long sought to explain how coexistence can arise through spatial competition among species. While the full answer certainly involves several axes of variation (Amarasekare 2003), many theorists have focused on individual mechanisms to improve our understanding of coexistence. One simple theory suggests that two species can coexist through a competition-colonization trade-off wherein one species is superior at colonizing unoccupied space while the other species is capable of displacing the former from occupied patches (Levins and Culver 1971).

The Levins-Culver model exhibits such a competition-colonization trade-off for two populations occupying the same niche and can be summarized by the system of equations:

7 $$\begin{aligned} \begin{aligned} \frac{dy}{dt}&= \beta _y y \left( 1-y\right) - \gamma _y y, \\ \frac{dz}{dt}&= \beta _z z\left( 1 - y - z\right) - \beta _y y z - \gamma _z z , \end{aligned} \end{aligned}$$

where *y* is the fraction of the niche occupied by species 1 and *z* is the fraction of the niche occupied by species 2 (Yu and Wilson 2001). The colonization rates of species 1 and 2 are limited by the availability of resources, represented by the terms $1-y$ and $1-y-z$, respectively. However, species 1 is able to colonize any area currently occupied by species 2.

At first glance, it may seem that species 2 will always be excluded from the niche through replacement by species 1. Through a stability analysis, it can be shown that if the intrinsic colonization rate of species 2, $\beta _z$, is large enough, then the two species can coexist in the long term. More precisely, if $\beta _z > \beta _y \left( \beta _y + \gamma _z - \gamma _y\right) /\gamma _y$ and $\beta _y > \gamma _y$, then the coexistence equilibrium, which satisfies

8 $$\begin{aligned} \begin{aligned} y&= 1-\frac{\gamma _{y}}{\beta _{y}}, \\ z&= \frac{\gamma _{y}}{\beta _{y}}-\frac{1}{\beta _{z}}\left( \gamma _{z}+\beta _{y}-\gamma _{y}\right) , \end{aligned} \end{aligned}$$

is locally asymptotically stable.

To finish this section, we give a proof that if $\beta _z > \beta _y \left( \beta _y + \gamma _z - \gamma _y\right) /\gamma _y$ and $\beta _y > \gamma _y$, then there is a locally asymptotically stable coexistence equilibrium using routing functions. The idea is to first construct the region boundaries in parameter space as described in Section 2.1, and then it is enough to check there is a locally asymptotically stable coexistence equilibrium at a single routing point in each region.

First, we construct the boundaries. The ideal ${\mathcal {I}}_f$ in this case is determined from the right-hand sides of equation (7)

$$ \langle \beta _y y \left( 1-y\right) - \gamma _y y, \beta _z z \left( 1 - y - z\right) - \beta _y y z - \gamma _z z \rangle . $$

The ideal is not prime; hence, we consider a primary decomposition which takes the form ${\mathcal {I}}_f = \bigcap _{i=1}^4 {\mathcal {J}}_f^{(i)}$ where

$$\begin{aligned} {\mathcal {J}}_f^{(1)}&= \langle y, z\rangle \\ {\mathcal {J}}_f^{(2)}&= \left\langle \beta _y\left( 1-y\right) -\gamma _y,z\right\rangle \\ {\mathcal {J}}_f^{(3)}&= \left\langle y,\beta _z\left( 1-z\right) -\gamma _z\right\rangle \\ {\mathcal {J}}_f^{(4)}&= \left\langle \beta _y\left( 1-y\right) -\gamma _y,\beta _z\left( 1-y-z\right) -\beta _y+\gamma _z\right\rangle \end{aligned}$$

The characteristic polynomial of the system is of the form $\lambda ^2 + a_1\lambda + a_0$ where $a_0$ and $a_1$ are below.

$$\begin{aligned} a_0&= 2y^{2}\beta _y^{2}+2y^{2}\beta _y\beta _z+4yz\beta _y\beta _z - y\beta _y^{2}-3y\beta _y\beta _z \\&\;\;\;\;\,-2z\beta _y\beta _z+y\beta _y\gamma _y+y\beta _z\gamma _y+2z\beta _z\gamma _y \\&\;\;\;\;\,+2y\beta _y\gamma _z+\beta _y\beta _z-\beta _z\gamma _y-\beta _y\gamma _z+\gamma _y\gamma _z\\ a_1&= 3y\beta _y+y\beta _z+2z\beta _z-\beta _y-\beta _z+\gamma _y+\gamma _z \end{aligned}$$

In order to find equations for the region boundaries, we need to compute the following elimination ideals and intersect them:

$$\begin{aligned}&({\mathcal {J}}_f^{(1)} + \langle a_0 a_1\rangle ) \cap \mathbb {C}[\beta _y,\beta _z,\gamma _y,\gamma _z], \\&({\mathcal {J}}_f^{(2)} + \langle y a_0 a_1\rangle ) \cap \mathbb {C}[\beta _y,\beta _z,\gamma _y,\gamma _z], \\&({\mathcal {J}}_f^{(3)} + \langle z a_0 a_1\rangle ) \cap \mathbb {C}[\beta _y,\beta _z,\gamma _y,\gamma _z], \\&({\mathcal {J}}_f^{(4)} + \langle y z a_0 a_1\rangle ) \cap \mathbb {C}[\beta _y,\beta _z,\gamma _y,\gamma _z]. \end{aligned}$$

The resulting ideal is principal with the total boundary defined by the vanishing of the product of the following polynomials:

$$\begin{array}{l} g_1 = \beta _y-\gamma _y, ~~~~ g_2 = \beta _z-\gamma _z, ~~~~ g_3=\beta _y-\beta _z-\gamma _y+\gamma _z,\\ g_4 = \beta _y+\beta _z-\gamma _y-\gamma _z, \\ g_5 = \beta _z\gamma _y-\beta _y\gamma _z, ~~~ g_6 = \beta _y^{2}-\beta _y\gamma _y-\beta _z\gamma _y+\beta _y\gamma _z,\\ g_7 = 2\,\beta _y^{2}-2\,\beta _y\gamma _y-\beta _z\gamma _y+\beta _y\gamma _z. \end{array}$$

Note that $g_1$ is the boundary of $\beta _y>\gamma _y$ and $g_6$ is the boundary of $\beta _z > \beta _y(\beta _y+\gamma _z-\gamma _y)/\gamma _y$.

Since we are only interested in parameter values that are positive, we include $\beta _y,\beta _z,\gamma _y,$ and $\gamma _z$ as boundaries along with $g_1,\dots ,g_7$ above, so the resulting arrangement ${\mathcal {A}}$ consists of 11 hypersurfaces. The routing function then takes the form

$$ r_c = \frac{\beta _y \cdot \beta _z\cdot \gamma _y\cdot \gamma _z\cdot \prod _{i=1}^7 g_i}{\left( 1 + (\beta _y - c_1)^2 + (\beta _z - c_2)^2 + (\gamma _y - c_3)^2 + (\gamma _z - c_4)^2\right) ^8} $$

For a problem of this size, the Julia package HypersurfaceRegions.jl automates the construction of the routing function as well as finding routing points and following gradient paths. We find that there are 168 regions in total; however, there are only 16 regions that divide up the positive orthant $\mathbb {R}_{>0}^4$. These are the 16 regions we need to test.

Of these 16 regions, there are 2 that make up the region where $\beta _z > \beta _y \left( \beta _y + \gamma _z - \gamma _y\right) /\gamma _y$ and $\beta _y > \gamma _y$. We can pick a representative routing point from each of these regions and check to see if these parameter values give rise to a locally asymptotically stable coexistence equilibrium. The parameter values are

$$\begin{aligned} (\beta _y,\beta _z,\gamma _y,\gamma _z)&= (1.03941, 1.76600, 0.93685, 0.22883),\\ (\beta _y,\beta _z,\gamma _y,\gamma _z)&= (1.21871, 1.48986, 0.75558, 0.23395). \end{aligned}$$

It is straightforward to check that each gives rise to a locally asymptotically stable coexistence equilibrium; therefore, every point in these regions gives rise to a stable equilibrium as this property can only change by crossing a boundary. On the other hand, if we check any of the other 14 regions, we find that these parameter values never give rise to a locally asymptotically stable coexistence equilibrium confirming the result stated earlier.

## Tripartite Symbiosis

In a recent article, Gibbs et al., formulated a model of coral-bacteria symbioses by extending the Levins-Culver model (Gibbs et al. 2024). In their model, the patch landscape, in this case, the coral reef available for bacteria to colonize, is no longer fixed but dynamically changing over time. In addition, one type of bacteria is assumed to increase the reproduction rate of the coral it colonizes (*mutualism*), while the other type of bacteria increases coral mortality (*parasitism*).

The coral populations are assumed to inhabit a reef with a total carrying capacity scaled to one. In the absence of bacteria, the unoccupied host coral population, *x*, grows over bare reef at the intrinsic per-capita growth rate *b* and dies off at the mortality rate *d*. The growth of the coral is inhibited by density dependence so that its size cannot exceed the carrying capacity of the bare reef.

The state variables *y* and *z* correspond to the amount of coral occupied by parasitic and mutualistic bacteria, respectively. These parasitic and mutualistic bacteria populations colonize coral through mass-action interaction with contact rates $\beta _y$ and $\beta _z$, respectively. The two types of bacteria cannot occupy the same patch of coral, hence $x+y+z\le 1$. Both bacterial types are obligate symbionts of the coral host, so their carrying capacities are limited by the available coral area. Additionally, if the coral they occupy dies off (which occurs at rate *d*), the bacteria die off as well. Due to virulence, the coral occupied by parasitic bacteria endures additional mortality at the rate $\tilde{d}$, which we refer to as the “death detriment” for brevity. Bacteria also die off on their own, leaving the coral it previously inhabited unoccupied, at rates $\gamma _y$ or $\gamma _z$. The total mortality rate for parasitic bacteria is, therefore, $d + \tilde{d} +\gamma _y$. Parasitic bacteria also colonize coral that is already occupied by mutualistic bacteria at the per-capita rate $\beta _y y$, thereby limiting the carrying capacity of mutualistic bacteria to $1-x-y$. Finally, colonization by the mutualistic bacteria induces an increased growth rate in the coral, $\tilde{b}$, which we refer to as “birth benefit” for brevity.

**Fig. 2:**
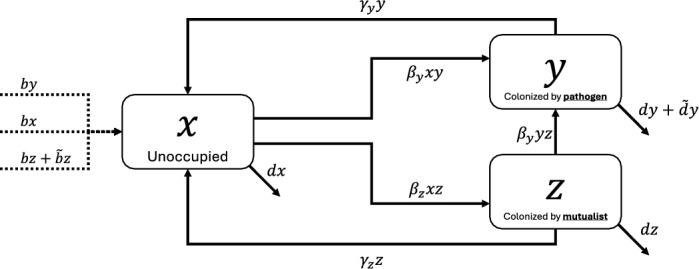
The coral-bacteria symbioses compartmental model represented by system (9)

The full system of ordinary differential equations describing this model is given by equations (9) and illustrated in Figure 2:

9 $$\begin{aligned} \begin{aligned} \frac{dx}{dt}&=[b(x+y)+(b+\tilde{b})z]\left[ 1-\left( x+y+z\right) \right] -\beta _{y}xy-\beta _{z}xz+\gamma _{y}y+\gamma _{z}z-dx,\\ \frac{dy}{dt}&=\beta _{y}xy+\beta _{y}yz-(d+\tilde{d}+\gamma _{y})y,\\ \frac{dz}{dt}&=\beta _{z}xz-\beta _{y}yz-\left( d+\gamma _{z}\right) z. \end{aligned} \end{aligned}$$

Of interest are the criteria (i.e., sets of parameters and initial conditions) for which i) coexistence among all three populations is stable; ii) mutualistic or parasitic bacteria are excluded from the system in the long term; iii) mutualistic or parasitic bacteria are able to invade a system where it is initially absent. Studying the criteria for coexistence or exclusion provides insights into the key drivers for the assembly of stable tripartite symbioses with competing symbionts. Such knowledge could inform the development of conservation strategies for coral reefs affected by acidifying and warming oceans by providing the criteria in which beneficial bacteria stably coexist with coral, with or without the presence of harmful bacteria.

We characterize the eight possible equilibria of system (9) into five types:

- total extinction equilibrium $E_0 = \left( 0,0,0\right)$;
- bacteria-free equilibria $E_x = \left( +,0,0\right)$, i.e. equilibria with a positive *x*-value and zero *y*- and *z*-values;
- mutualistic bacteria exclusion equilibria $E_{xy} = \left( +,+,0\right)$: equilibria with positive *x*- and *y*-values and zero *z*-value;
- parasitic bacteria exclusion equilibria $E_{xz} = \left( +,0,+\right)$: equilibria with positive *x*- and *z*-values and zero *y*-value; and
- full coexistence equilibria $E_{\text {co}} = \left( +,+,+\right)$: equilibria with all positive coordinate values.

The results of Gibbs et al. (2024) determined criteria for the existence and stability of these equilibria under the assumption that the mutualistic bacteria had no effect on the growth of the coral ($\tilde{b}=0$) as well as when both bacteria had no effect whatsoever on coral life history ($\tilde{b}=0$ and $\tilde{d}=0$). The other cases were considered numerically, where it was discovered that stable limit cycles around the coexistence equilibrium point could occur for certain parameter combinations. However, a full description of the stability landscape when $\tilde{b}$ and $\tilde{d}$ were both positive was not provided.

We originally sought to fully describe the equilibrium dynamics of (9) by utilizing the techniques outlined in Section 2.2 just as was accomplished for for the Levins-Culver model in Section 3. However, due to the large the number of parameters, the Gröbner basis computations for computing the boundaries did not finish for the entire model. Thus, to reduce to number of parameters, we set all the death rates equal to 1 and the birth rate of the parasitic bacteria is set to 5, i.e. $d = \gamma _y = \gamma _z = 1$ and $\beta _y = 5$. The resulting model has four free parameters: $b, \beta _z, \tilde{b},$ and $\tilde{d}$.

Proceeding as in the end of Section 3, we compute the equations defining the total boundary. It turns out that the ideal ${\mathcal {I}}_f \subset \mathbb {C}[x,y,z,b,\beta _z,\tilde{b},\tilde{d}]$ in this case has 5 primary components, one corresponding to each of the five equilibria state types: $E_0, E_x, E_{xy}, E_{xz}, E_\text {co}$. After adding in the Routh-Hurwitz conditions and eliminating *x*, *y*, *z*, we are left with a principal ideal $\langle g \rangle \subset \mathbb {C}[b,\beta _z,\tilde{b},\tilde{d}]$. The polynomial *g* has 31 distinct irreducible factors, $g_1,\dotsc ,g_{31}$, whose degrees range from 1 to 20. In fact, there are 6 linear factors, 7 quadratic factors, 2 cubic factors, 4 quartic factors, 4 quintic factors, 3 degree 9 factors, and the remaining 5 factors have degrees 6, 7, 10, 13, and 20. We end up with the total boundary ${\mathcal {B}} = \bigcup _{i=1}^{31} \{a \in \mathbb {R}^4 ~|~ g_i(a) = 0\}$. These computations were done in Macaulay2 (Grayson and Stillman 2022) and can be found in a GitHub repository.

The next step is to compute the full set of routing points where each connected component of the complement of the boundary in $\mathbb {R}^4_{>0}$ contains a single routing point. Since the degree of the numerator of the routing function, namely $g_1\cdots g_{31}$, has degree 145, computing the routing points in full generality was numerically unstable. Thus, we consider two major cases: when $b = 2$ and when $b = 0.5$. These values were chosen because they correspond to cases where the coral can persist on its own ($b=2>d=1$) and where it cannot ($b=0.5<d=1$). Correspondingly, $b - 1$ is one of the six linear factors in *g*. For each of these values of *b*, we consider the stability landscape for increasing values of the colonization rate of the mutualistic bacteria: $\beta _z = 2.5, 3, 3.5, 3.9, 4.1, 5.9, 6.1$ and 10. The values 3.9, 4.1, 5.9 and 6.1 were chosen since when $b = 2$, the linear polynomials $\beta _z - 4$ and $\beta _z - 6$ are factors of $g\vert _{b=2}$.

For each of these 16 slices, we construct a routing function and compute the connected components of $\mathbb {R}^2_{>0} \setminus {\mathcal {B}}$. Explicitly, for a given slice, $b = s_1$ and $\beta _z = s_2$, the routing function takes the following form:

$$ r_c(\tilde{b}, \tilde{d}) = \frac{g(s_1,s_2,\tilde{b},\tilde{d})}{(1 + (\tilde{b} - c_1)^2 + (\tilde{d} - c_2)^2)^D} $$

for a random choice of $(c_1,c_2) \in \mathbb {R}^2$ and $2D > \deg (g)$. Also, note that *g* contains the coordinate boundaries by construction, aligning with our set up in Section 2.2. A routing point in each connected component was chosen and the possible steady states for these parameter values were computed. Finally, we colored each region according to which steady states were possible at the corresponding routing point. These computations were done using the Julia package HomotopyContinuation.jl (Breiding and Timme 2018). The results of these computations can be found in the next section, and all computations are freely available in a GitHub repository.

### Simulations

The following analyzes the three free parameters: the birth benefit of mutualist bacteria to the coral host ($\tilde{b}$), the death detriment imposed by parasitic bacteria to the coral host ($\tilde{d}$), and the colonization rate of mutualistic bacteria ($\beta _z$). To focus on the effects of the symbionts on their host (via $\tilde{b}$ and $\tilde{d}$), our results are presented as “slices” along the $\beta _z$ axis. The names and assigned colors for each stable steady state set are presented in Table 2.

**Table 2:** Color key for stability landscape labels

**Label**	**Name**	**Color**
$E_0 = (0,0,0)$	Total extinction	
$E_x = (+,0,0)$	Coral only	
$E_{xy} = (+,+,0)$	Mutualist exclusion	
$E_{xz} = (+,0,+)$	Pathogen exclusion	
$E_{co} = (+,+,+)$	Coexistence	
$S_{0,xy} = \left\{ E_0, E_{xy}\right\}$	Extinction or pathogen exclusion	
$S_{0,co}=\left\{ E_0, E_{co}\right\}$	Extinction or coexistence	
$S_{x,xz} = \left\{ E_x, E_{xz}\right\}$	Coral only or pathogen exclusion	
$S_{x,co} = \left\{ E_x, E_{co}\right\}$	Coral only or coexistence	

#### Intrinsically Viable Coral Population ($b=2$)

**Fig. 3:**
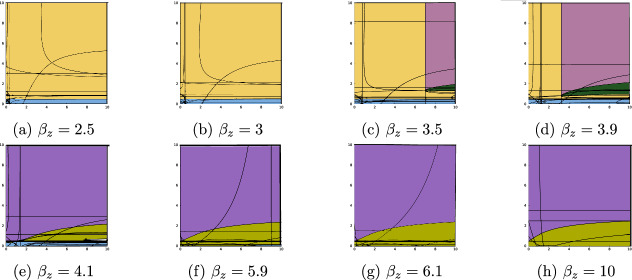
Stability landscapes for increasing values of $\beta _z$ with $b = 2$. In this case, the intrinsic coral birth rate *b* exceeds the intrinsic coral mortality rate $d=1$. $\tilde{b}$ values are varied along the *x*-axis and $\tilde{d}$ values along the *y*-axis from zero to ten (Color figure online)

Figure 3 shows the changes in the $b=2$ stability landscape as $\beta _z$ is increased from 2.5 to 10. At low values of $\beta _z$, the coral only steady state $E_x$ is stable, with a very narrow stability region for mutualist exclusion $E_{xy}$ at low levels of death detriment $\tilde{d}$. As $\beta _z$ is increased to 3.5, there is bistability for sufficiently large values of reproductive benefit, $\tilde{b}$, and mortality detriment, $\tilde{d}$. The mutualist population is positive only within these regions of bistability, $S_{x,xz}=\{E_x,E_{xz}\}$ and $S_{x,co}=\{E_x,E_{co}\}$. As $\beta _z$ crosses four, the stable sets become $E_{xy}$, $E_{xz}$, and $E_{co}$ and bistability is no longer possible. Across most of the landscape, the pathogen is now excluded, with a very narrow region of mutualist exclusion for low $\tilde{d}$, a small region where coexistence is stable, and a very small region where no steady states are stable (shown in white). As $\beta _z$ is further increased, the region of mutualistic extinction vanishes, and the coexistence region grows slightly.

In this case, the extinction state $E_0$ is not stable for any combination of $\beta _z$, $\tilde{b}$, and $\tilde{d}$ since the coral can persist without the assistance of the mutualistic bacteria. For our chosen $\beta _z$ values, bistability only occurred for values of $\beta _z=3.5$ and 3.9. When $\beta _z=4.1$, there is a small white region in (e) for which none of the steady states are stable and the model must have a limit cycle. For any value of $\beta _z$, a stable steady state can only contain parasitic bacteria if $\tilde{d}$ is sufficiently low, with an upper limit of approximately $\tilde{d} = 2.5$ across all values of $\beta _z$.

**Fig. 4:**
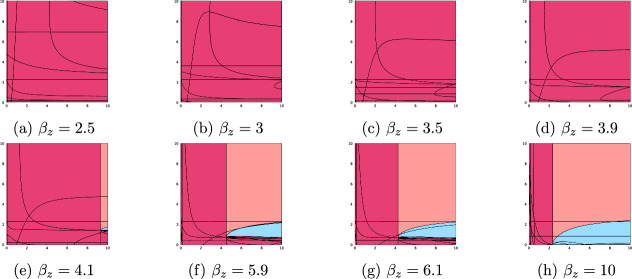
Stability landscapes for increasing values of $\beta _z$ with $b = 0.5$. In this case, the intrinsic coral birth rate *b* is less than the intrinsic coral mortality rate $d=1$. $\tilde{b}$ values are varied along the *x*-axis and $\tilde{d}$ values along the *y*-axis from zero to ten (Color figure online)

#### Intrinsically Non-Viable Coral Population ($b=0.5$)

Figure 4 shows the changes in the $b=0.5$ stability landscape as $\beta _z$ is increased from 2.5 to 10. In this case, where $b = 0.5 < d = 1$, all stable steady state sets include the total extinction steady state $E_0$. Total extinction may be paired with pathogen exclusion, $S_{0,xz} = \left\{ E_0,E_{xz}\right\}$, or coexistence, $S_{0,co} = \left\{ E_0,E_{co}\right\}$, but never mutualist exclusion. In this frame of $\tilde{b}$ and $\tilde{d}$ values, non-extinction steady states were only stable when $\beta _z$ exceeds four (Figure 4, (e)-(h)), that is, when the ability of the mutualistic bacteria to colonize coral was sufficiently high. In this case, the non-extinction steady states $E_{xz}$ or $E_{co}$ could only be stable if $\tilde{b}$ was sufficiently large, with this breakpoint decreasing as $\beta _z$ increases. If $\tilde{d}$ exceeded approximately 2.5, the coexistence steady state could not be stable (e-h). Surprisingly, for $\beta _z$ between 4.1 and 6.1, there is also a lower bound for $\tilde{d}$ below which the coexistence steady state $E_{co}$ cannot be stable. However, as $\beta _z$ is increased to ten (Figure 4 (h)), this lower bound vanishes.

### Summary of Results

Increasing the transmissibility of the mutualistic bacteria ($\beta _z$) caused a transformation in the stability landscape, whether the coral host is intrinsically viable ($b>d$) or not ($b<d$). When the coral host was intrinsically viable, increasing $\beta _z$ led to a shift in the landscape from one where the coral only steady state $E_x$ was almost always stable (with the possibility of bistability), to one where coral populations always exist and all stable steady states include one of the colonized coral subpopulations. In particular, the region of bistability $S_{x,co}$, seen when $\beta _z = 3.9$, abruptly becomes a region of stable coexistence as $\beta _z$ is increased beyond four. This suggests that the mutualistic bacteria can facilitate coexistence with the parasitic bacteria through their ability to colonize coral. However, stable coexistence always requires that the detrimental effect of the parasitic bacteria ($\tilde{d}$) remains below a certain threshold.

When the coral population was not intrinsically viable ($b<d$), the possibility of viability emerged when the transmissibility of the mutualistic bacteria ($\beta _z$) exceeded four. Still, total extinction was always a stable steady state, no matter the value of $\beta _z$. This suggests that if environmental perturbations cause shifts in coral biology leading to their intrinsic inviability, these populations could be preserved if mutualistic bacteria are present. Since these regions are bistable, abundances likely must remain high to avoid landing in the stable manifold for total extinction. Thus, shocks to populations, such as extreme weather events, could shift stable regimes to ones of total extinction.

## Conclusion

State transitions are ubiquitous in nature, and dynamic models are commonly used to understand how perturbations or interventions can induce or prevent such transitions. Descriptions of stability landscapes allow for the determination of parameter regimes wherein a desired future state is stable or an unfavored state is unstable. Mapping parameter spaces into stability landscapes requires evaluation of a system of polynomial equations and inequalities in several variables, a problem that is often analytically and numerically intractable.

The routing function method and algorithm presented here provide a straightforward manner of conducting steady-state analysis and numerically determining stability landscapes. Routing functions were originally proposed in Hong (2010) for answering connectivity queries and extended to computing smoothly connected components in Cummings et al. (2025). The proposed extension to analyze stability landscapes involves four steps: (1) compute the boundary ideal, (2) construct a routing function and compute its critical points, (3) track gradient descent/ascent paths from high index critical points to lower index critical points, and (4) analyze the possible steady states in each connected component in the complement of the boundary. The utility of this method is complemented by the availability of computational tools to symbolically compute boundary ideals and to numerically compute critical points and track paths, particularly for the complement of hypersurfaces.

In this work, we illustrated the use of this method through the analysis of the coral-bacteria symbiosis model (Gibbs et al. 2024). While this model is relatively simple, describing the entire stability landscape across the five types of equilibria proved intractable using standard methods. After fixing certain parameters, the routing function method enabled us to further elaborate on the conditions under which bacterial colonization supports the persistence of coral populations. In particular, we determined that colonization by mutualistic bacteria can facilitate the persistence of coral populations, whether or not the coral population is viable on its own. In addition, we identified regions of bistability that can be used to determine whether shocks to populations might transition them from healthy, stable states to total extinction. This method also allowed us to identify a region with no stable steady states for which the system must admit limit cycles or more complex attractors.

For simplicity, we restricted our example to explore the stability landscape induced by certain rate parameters. This allowed for a rough, qualitative sensitivity analysis of various stable steady states as these two parameters varied within a given “frame.” While this provided a broad theoretical picture of model dynamics, in real-world applications, the parameters treated as variables derive from the available data (or lack thereof) and the particular research question. Computational efficiency of the routing function method depends on the feasibility of symbolically computing boundary ideals and numerical conditioning of the routing points and gradient descent/ascent paths. Of course, using two or three parameters as variables is often the best choice when the goal is to produce results that can be visually interpreted.

While not considered here, the routing function method can be used to address a range of other questions commonly pursued with stability analysis. For example, given the existence of bistability or, more generally, *k*-stability, one can treat initial conditions as parameters to determine the stability landscape with respect to the initial conditions. Connectivity questions can also be addressed, which was the original motivation for developing routing functions. As demonstrated, this method is particularly well-suited to address common problems in the analysis of ecological models, in particular the description of stability landscapes. The adoption of this method by mathematical biologists and ecologists has the potential to expand the range of systems that can be analyzed and improve the understanding of parameters and their impact on the behavior of the model.

## Data Availability

All data and code necessary to replicate the results in this article are available at https://github.com/jcu237/Routing-functions-for-parameter-space-decomposition-to-describe-stability-landscapes.

## A Appendix

The Routh-Hurwitz criterion gives necessary and sufficient conditions for a polynomial with real coefficients to have all its roots lie in the left-half plane $\{z \in \mathbb {C} ~|~ Re(z) <0\}$. In this appendix, we will describe the stability criterion through the leading principal minors of the Hurwitz matrix. This is different (but equivalent) to the so-called Routh array. Throughout, we will let

$$ f = a_n s^n + a_{n-1}s^{n-1} + \dotsc + a_1s + a_0 \in \mathbb {R}[s] $$

Consider the $n\times n$ matrix given by

$$ H = \begin{pmatrix} a_{n-1} & a_{n-3} & a_{n-5} & \dots & \dots & \dots & 0 \\ a_n & a_{n-2} & a_{n-4} & \dots & \dots & \dots & 0 \\ 0 & a_{n-1} & a_{n-3} & \dots & \dots & \dots & 0\\ 0 & a_n & a_{n-2} & \dots & \dots & \dots & 0\\ \vdots & \vdots & \vdots & \ddots & \ddots & \ddots & \vdots \\ 0 & 0 & \dots & a_{2\lfloor n/2\rfloor } & \dots & a_{2} & a_0 \end{pmatrix}. $$

Let $\Delta _i$ be the $i^\text {th}$ leading principal minor of *H*, i.e. the determinant of the $i\times i$ submatrix of *H* given by the first *i* rows and columns of *H*. The proof of the following theorem can be found in Gantmacher (1959, Vol. 2, Chapter XV).

### Theorem A.1

(The Routh-Hurwitz Stability Criterion) Let $f = \sum _{i=0}^n a_i s^i$ be a polynomial of degree *n* with real coefficients, and assume without loss of generality that $a_n > 0$. The roots of *f* lie in the left half-plane, $\{z \in \mathbb {C} ~|~ Re(z) < 0\}$, if and only if $a_i > 0$ and $\Delta _i > 0$ for all *i*. In other words, *f* is stable if and only if it has positive coefficients and *H* is positive definite.

Below, we list these conditions explicitly for $n = 2,3,4$.

### Example 4

When $n = 2$, we have

$$ H = \begin{pmatrix} a_1 & 0 \\ a_2 & a_0 \end{pmatrix}. $$

Therefore, if $a_1> 0, a_0a_1 > 0$, $a_2 > 0$, all roots of *f* will have negative real part.

### Example 5

When $n = 3$, we have

$$ H = \begin{pmatrix} a_2 & a_0 & 0 \\ a_3 & a_1 & 0 \\ 0 & a_2 & a_0 \end{pmatrix}. $$

The leading principal minors are as follows.

$$\begin{aligned} \Delta _1&= a_2 \\ \Delta _2&= a_1 a_2 - a_0 a_3 \\ \Delta _3&= a_0 \Delta _2 \end{aligned}$$

Then all of the roots of $f = a_3 s^3 + a_2 s^2 + a_1 s + a_0$ lie in the left-half plane if and only if $a_0,a_1,a_2,a_3 > 0$ and $a_1 a_2 - a_0 a_3 > 0$.

### Example 6

When $n = 4$, we have

$$ H = \begin{pmatrix} a_3 & a_1 & 0 & 0\\ a_4 & a_2 & a_0 & 0\\ 0 & a_3 & a_1 & 0 \\ 0 & a_4 & a_2 & a_0 \end{pmatrix} $$

The leading principal minors are as follows.

$$\begin{aligned} \Delta _1&= a_3 \\ \Delta _2&= a_2 a_3 - a_1 a_4 \\ \Delta _3&= a_1a_2a_3 - a_0a_3^2 - a_1^2a_4 = a_1\Delta _2 - a_0 a_3^2 \\ \Delta _4&= a_0 \Delta _3 \end{aligned}$$

These must all be positive along with each $a_i$ in order for *f* to have all its roots lie in the left-half plane.
